# A Systematic Review of Disappearing Colorectal Liver Metastases: Resection or No Resection?

**DOI:** 10.3390/jcm14041147

**Published:** 2025-02-10

**Authors:** Menelaos Papakonstantinou, Antonios Fantakis, Guido Torzilli, Matteo Donadon, Paraskevi Chatzikomnitsa, Dimitrios Giakoustidis, Vasileios N. Papadopoulos, Alexandros Giakoustidis

**Affiliations:** 1 Aristotle University Surgery Department, Papageorgiou Hospital, Aristotle University of Thessaloniki, 54124 Thessaloniki, Greece; menelaospap.md@gmail.com (M.P.); antfandakis@yahoo.gr (A.F.); voula.hatzikomnitsa@gmail.com (P.C.); dgiakoustidis@gmail.com (D.G.); papadvas@auth.gr (V.N.P.); 2 Department of Surgery, Division of Hepatobiliary Surgery & General Surgery, Humanitas Research Hospital, 20089 Rozzano, Italy; guido.torzilli@hunimed.eu; 3 Surgical Oncology Program, University Maggiore Hospital, University of Piemonte Orientale, 28100 Novara, Italy; matteo.donadon@cancercenter.humanitas.it

**Keywords:** disappearing liver metastases, colorectal liver metastases, resection, recurrence

## Abstract

**Background:** Colorectal cancer is the second most common type of cancer and a leading cause of cancer-related deaths worldwide. Approximately 15% of the patients with colorectal cancer will already have liver metastases (CRLMs) at diagnosis. Luckily, the advances in chemotherapy regimens during the past few decades have led to increased rates of disease regression that could even render an originally unresectable disease resectable. In certain patients with CRLMs, the hepatic lesions are missing on preoperative imaging after neoadjuvant chemotherapy. These patients can undergo surgery with or without resection of the sites of the disappearing liver metastases (DLMs). In this systematic review, we assess the recurrence rate of the DLMs that were left unresected as well as the complete pathologic response of those resected. **Methods:** A literature search was conducted in PubMed for studies including patients with CRLMs who received neoadjuvant chemotherapy and had DLMs in preoperative imaging. Two independent reviewers completed the search according to the PRISMA checklist. **Results:** Three hundred and twenty-six patients with 1134 DLMs were included in our review. A total of 47 out of 480 DLMs (72.29%) that were removed had viable tumor cells in postoperative histology. One hundred and forty-five tumors could not be identified intraoperatively and were removed based on previous imaging, with thirty (20.69%) of them presenting viable cancer cells. Four hundred and sixty-five lesions could not be identified and were left in place. Of them, 152 (32.69%) developed local recurrence within 5 years. Of note, 34 DLMs could not be categorized as viable or non-viable tumors. Finally, DLMs that were identifiable intraoperatively had a higher possibility of viable tumors compared to non-identifiable ones (72.29% vs. 20.69%, respectively). **Conclusions:** Disappearing liver metastases that are left unresected have an increased possibility of recurrence. Patients receiving neoadjuvant treatment for CRLMs may have better survival chances after resecting all the DLM sites, either identifiable intraoperatively or not.

## 1. Introduction

### 1.1. Colorectal Cancer and Colorectal Liver Metastases

Colorectal cancer (CRC) is the second most frequent type of malignancy and, as a result, one of the leading causes of death worldwide. Approximately 15% of patients already have colorectal liver metastases on diagnosis, and approximately 16% will develop lesions within the first 5 years, accounting for one-third of the patients in total. Between 75% and 90% of CRC patients present with unresectable tumors at diagnosis [1,2,3,4,5,6]. Metastatic liver disease predicts poor prognosis with 5-year survival between 12% and 17% [7,8].

The advancement of medicine and pharmacology has led to new and more effective chemotherapeutic drugs that can greatly assist in the battle against cancer. And when they are combined with new surgical techniques and non-surgical interventional methods, such as portal vein embolization, radiofrequency and microwave ablation, as well as a two-stage hepatectomy, metastatic liver disease can be treated way more effectively, improving the 1- and 5-year survival by to up to 93% and 47%, respectively [9,10,11,12,13,14].

Although patients with resectable liver metastases have been treated surgically, the tendency of administering neo-adjuvant chemotherapy is gradually increasing with very good results and can even lead to a complete clinical response (CCR). In such cases, the liver metastases may not be detectable in the post-chemotherapy restaging, even during intraoperative imaging, which has raised conflict in the literature regarding the optimal management of disappearing liver metastases (DLMs) [10,13,15,16,17]. The disappearing liver metastases incidence ranges between 7% and 37% in up to 23% of patients with metastatic liver disease [1,10,13,18,19,20]. Additionally, chemotherapy can turn nonresectable tumors into resectable tumors in up to 40% of patients with liver metastases (LMs) [21,22,23,24,25]. However, the disappearing lesions do not always represent a complete pathological response, which varies from 15% to 73% and constitutes a strong predictor of survival. Since there is a chance for recurrence, which ranges from 4.9% to 27.2% within 2 years of follow-up, many authors suggest surgical exploration for the resection of all pre-chemotherapy liver lesions in order to improve survival [1,4,15,26,27,28,29]. This is why the combination of hepatic resection and systemic chemotherapy is becoming the gold standard for the treatment of metastatic colorectal cancer [30,31]. However, controversy still remains regarding the resection or not of metastatic lesions that are undetectable after neoadjuvant chemotherapy.

### 1.2. Lesion Detection

The methods used for the detection of LMs are computed tomography (CT), magnetic resonance tomography (MRI) and ultrasound (U/S), both pre- and post-chemotherapy. MRI has a reported sensitivity of 85% for detecting LMs after chemotherapy, which increases greatly after the administration of gadoxetic acid, a hepatocyte-specific contrast agent, allowing the detection of small colorectal LMs, which cannot be detected by CT or U/S [32,33,34]. During resection, the use of intraoperative ultrasonography (IOUS), which can be assisted by intravenous ultrasound contrast, is the main means of LM detection [10]. For disappeared lesions that could not be detected intraoperatively by IOUS, the pattern from previous imaging techniques can be used, with noticeable anatomic structures and detectable LMs being used as landmarks [35].

### 1.3. Aim of Study

The aim of this study is to assess the behavior and recurrence rate of unresected DLMs in patients with CRLMs in order to evaluate whether surgical treatment is beneficial for patient survival.

## 2. Materials and Methods

### 2.1. Search Strategy

**Figure 1:**
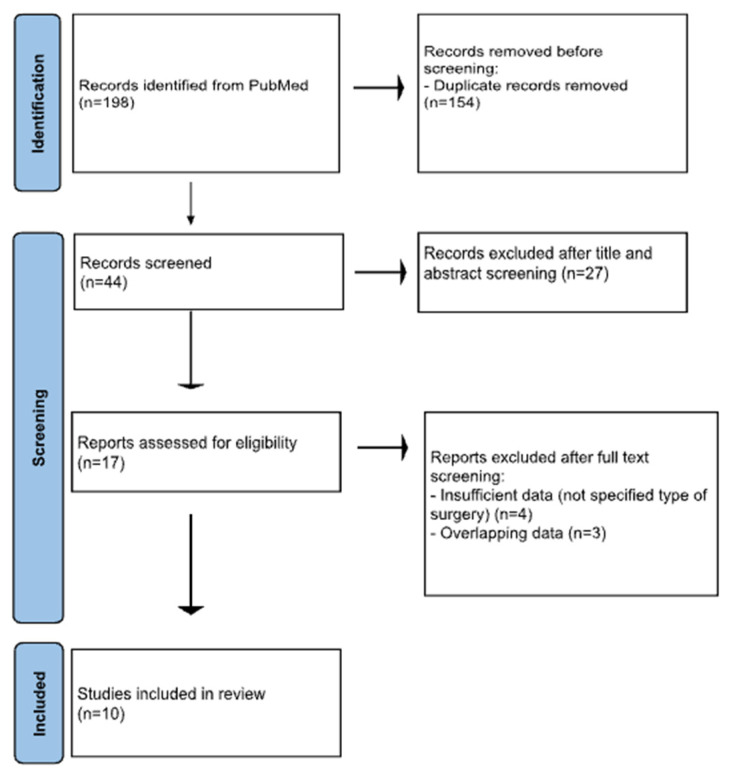
PRISMA flowchart.

A systematic literature search was performed in PubMed for all publications including patients with disappearing colorectal liver metastases. The terms “liver metastases”, “colorectal liver metastases”, “disappearing”, “missing”, “hepatectomy”, “surgery” and “management” were used interchangeably, and the search yielded 198 results. A hundred and fifty-four (154) duplicate records were removed, and 44 studies underwent further assessment. After title and abstract screening, 27 irrelevant articles were excluded, and 17 were eligible for full-text screening. Finally, 10 records were included in our systematic review [1,2,4,10,13,18,20,36,37,38]. Two independent reviewers (M.P. and A.F.) completed the search according to the PRISMA checklist (the PRISMA algorithm is shown in Figure 1). Any conflict during the selection process was resolved through discussion with a third reviewer (P.C.). Finally, the research protocol was registered and can be accessed at the International Prospective Register of Systematic Reviews (PROSPERO, ID CRD42024541813).

### 2.2. Inclusion and Exclusion Criteria

We included studies that met the following inclusion criteria: cohort studies including adult patients with colorectal liver metastases, studies in the English language, patients who underwent neoadjuvant chemotherapy and patients with at least one DLM in preoperative imaging.

Studies in a language other than English, case reports and case series were excluded from our systematic review.

### 2.3. Data Extraction

The following data were extracted to a preformed datasheet: year of publication, study period, institute, country, study type, population number, age, sex, primary tumor site, stage of the primary tumor, neoadjuvant and adjuvant chemotherapy regimens, number of liver metastases (LMs), preoperative and intraoperative imaging, type of hepatectomy, the response of LMs to chemotherapy, number of resected sites, histology of resected sites, the true complete response of LMs, cancer recurrence, recurrence-free and overall survival of the patients. Any missing or unclear information was identified as such and not included in our analysis.

### 2.4. Definition

A DLM was defined as a hepatic metastatic lesion present on the baseline imaging but unidentifiable on the post-chemotherapy restaging imaging (US, CT, MRI or PET/CT scan) regardless of the findings of IOUS.

### 2.5. Risk of Bias and Quality Assessment

We used the ROBINS-I tool to assess the risk of bias in each of the cohort studies included (Table 1) and the Newcastle–Ottawa scale for the quality assessment of the studies (Table 2) [39,40].

## 3. Results

Ten cohort studies were included in this systematic review regarding patients with DLMs after neoadjuvant therapy for metastatic colorectal cancer. The response to the chemotherapy was evaluated preoperatively via contrast-enhanced CT and MRI, as well as ultrasound and PET/CT (Table 3).

A total of 326 patients with 1134 DLMs were included. The patient demographics and preoperative characteristics are shown in Table 4. Four hundred and eighty lesions were identified intraoperatively by either IOUS or palpation and removed. A total of 47 out of 480 (72.29%) had viable tumor cells in postoperative histology (Table 5). In contrast, 145 tumors that could not be identified intraoperatively were removed based on previous imaging, and 30 (20.69%) of them presented viable cancer cells (Table 6). Four hundred and sixty-five lesions could not be identified and were left in place. Of them, 152 (32.69%) developed local recurrence within 5 years of follow-up (Table 7). Twenty-eight DLMs underwent radiofrequency ablation (RFA) and could not be categorized as viable or non-viable tumors, while six more lesions had intermediate pathology reports and were not categorized.

DLMs that were identifiable intraoperatively either by palpation or IOUS had a significantly higher possibility of being viable tumors compared to non-identifiable ones (72.29% vs. 20.69%). DLMs left in place presented a 32.69% chance of developing recurrence. Patients with complete pathological response had more favorable outcomes compared with those that had viable cancer at the site of DLM, as did patients who underwent surgery and had all the DLMs removed, in comparison with those who underwent partial excision.

## 4. Discussion

In this systematic review, we assessed patients with colorectal liver metastases that disappeared on preoperative imaging after neoadjuvant treatment. The DLMs were either resected or left in place and followed up. We found that the recurrence rate of unresected DLMs ranged from 14.3% to 74.19%, with a mean recurrence rate of 39.37%. Of note, even though some liver lesions were missing in the preoperative imaging, they were identified intraoperatively with either palpation or IOUS and removed. Up to 80% of undetected and removed DLMs harbored viable carcinoma, while the percentage of viable carcinoma of those detected and removed reached 100% in certain cases. Interestingly, a complete pathological response was as low as 20% in patients with disappearing lesions in preoperative imaging. This finding highlights the significance of intraoperative modalities, as the preoperative imaging is only suggestive of the response to chemotherapy and should not guide the final decision-making.

Our results are in line with those of other literature reviews. For instance, in the study by Araujo et al., 201 of 706 DLMs were not detected via imaging and left unresected, and 60 (29.8%) had local recurrence. Of note, 80% of the resected sites revealed residual disease, even though the lesions were radiographically unidentifiable [41]. Similar results were reported by Bischof et al.; hence, both Araujo et al. and Bischof et al. suggested resecting all DLM sites [32,41]. Their conclusion is consistent with an expert consensus on CRLMs that proposed resecting all metastatic sites, including those that were present before chemotherapy [42]. However, a recent Italian survey revealed that there is no standard approach to managing DLMs (to resect or not resect), even among specialized hepatobiliary surgeons [43]. In 2021, the consensus of the Japanese Society of Hepato-Biliary-Pancreatic Surgery recommended hepatic resection for CRLMs that are smaller than 3cm; however, the resection of the disappeared lesions was not specifically addressed, and the level of evidence was low [44]. Nonetheless, since the reported recurrence rate of unresected DLMs is high, the current literature data favor the resection of all DLMs, either identifiable intraoperatively or not, in order to improve disease-free survival. In the future, high-level-of-evidence studies, such as randomized controlled trials, should be conducted in large-volume centers to guide and standardize clinical practice regarding the management of DLMs and potentially optimize the treatment of patients.

The response to neoadjuvant chemotherapy is critical in the management of both unresectable and initially resectable CRLMs. Unfortunately, it is still impossible to predict a true complete response since a complete clinical/radiological response may not entail a complete pathological response [45,46]. For that reason, Zalinski et al. suggest repeating imaging after a few chemotherapy cycles to mark lesions about to disappear and then safely resect or ablate the DLM sites [47]. FOLFIRINOX is the cornerstone of therapy, but the regimen is adjusted based on a patient’s clinical status. Adding monoclonal antibodies (cetuximab or panitumumab) in patients with CRLMs and RAS or BRAF mutations could enhance the elimination of liver metastases, but the available data are conflicting [48]. For instance, in the CELIM clinical trial, cetuximab increased the therapeutic effect of the standard regimen [11]. However, in another large clinical trial (TRIPLETE trial), panitumumab’s benefit did not exceed the gastrointestinal adverse events [49].

The NCCN guidelines recommend a CT scan following neoadjuvant chemotherapy in order to assess the response to the therapy [50]. The patients included in our study had a CT, an MRI scan or a US before surgery as a means to detect possible DLMs. It is important to mention that after chemotherapy, there is a possibility of developing liver steatosis. In that setting, an MRI represents the most accurate imaging modality and should be used to complement the first-line CT scan [51,52,53]. In a study by VanKessel et al., the MRI sensitivity for CRLM detection was 85.7%, while only 69.9% for CT and 54.5% for the PET scan [54]. The ability of the MRI to detect small liver lesions is even greater when novel liver-specific contrast agents, such as gadolinium ethoxy benzyl dimeglumine (Gd-EOB-DTPA) or gadoxetate disodium, are used, which may even alter the surgical planning [55,56,57]. However, the most reliable method to detect metastatic lesions is by IOUS and contrast-enhanced IOUS, which has a sensitivity of about 97% and allows the detection of lesions that were initially missed on preoperative imaging. Moreover, IOUS can be fused with CT or MRI images, which may lead to the increased intraoperative identification of missing LMs [10,41].

Advancements in radiology could make the identification of missing liver metastases on preoperative imaging after neoadjuvant chemotherapy even more accurate and easy. Radiologists can recognize lesions at high risk of disappearing during preoperative multidisciplinary assessment. Placing a fiducial marker at the site of a DLM high-risk lesion before chemotherapy would eliminate the risk of leaving a DLM site unresected [58,59]. Due to the possible complications, a fiducial marker should be considered only in CRLMs at high risk of disappearance, such as lesions of less than 25 mm in diameter and 10mm in depth into the liver parenchyma [58]. On the contrary, 3D modeling helps to recognize the positioning of a lesion relative to nearby vessels, allowing the surgeon to locate the lesion more easily in the operating room without the complication risk of any intervention [59]. Finally, the evolution of radiomics and the integration of artificial intelligence and machine learning in the identification and prognosis of liver metastases has the potential to bring revolution in surgical oncology [60]. For instance, there are machine learning algorithms that have the ability to predict the survival of patients with CRLMs or the response to chemotherapy by analyzing CT images [61,62,63]. This technology is still preliminary and not widely used in clinical practice; however, it could provide crucial guidance in decision-making regarding the therapeutic approach to patients with liver metastases based on the preoperative restaging images and, therefore, avoid any non-beneficial interventions.

One limitation of our study is that the DLMs were identified based on preoperative and intraoperative imaging modalities with variable sensitivity. Some of the included studies used only CT scans to detect DLMs preoperatively, while others used both CT, MRI and US. The degree of hepatic steatosis after chemotherapy, the depth of the disappearing lesion into the liver parenchyma and its location in relation to anatomical landmarks could all possibly affect the ability to detect a DLM intraoperatively. In addition, the sensitivity of IOUS in the recognition of a DLM depends on the operator; consequently, the experience and the skill of the surgeon in using IOUS could also affect the results. For instance, a missed DLM intraoperatively that has not truly disappeared could lead to a higher reported recurrence rate. Additionally, the resection of the DLMs was performed by different surgeons and various surgical techniques in independent institutions around the world, which increases the bias. Finally, variable chemotherapy regimens were administered to the patients, which may also lead to a discrepancy in recurrence.

## 5. Conclusions

In conclusion, our systematic review shows that the recurrence of CRLMs was lower in patients with DLMs that were resected than in those left unresected. Therefore, resecting the sites of the disappearing lesions should be considered since it may offer better survival chances. Advances in radiology are going to evolve preoperative and intraoperative imaging so that surgeons can easily identify and safely resect completely disappeared lesions. However, there are no standard guidelines for the management of patients with CRLMs that disappear after neoadjuvant chemotherapy. Future research should focus on prospective studies in large-volume centers in order to present robust data and suggest a safe and effective protocol for managing patients with DLMs.

## Figures and Tables

**Table 1:** The ROBINS-I tool.

Study	Confounding Bias	Selection Bias	Bias in Classification of Interventions	Bias Due to Deviation from Intended Interventions	Bias Due to Missing Data	Bias in Measurement of Outcomes	Bias in Selection of the Reported Result
Ferrero A et al. [10]	moderate	low	low	low	low	moderate	low
Kim SS et al. [4]	moderate	low	low	low	moderate	low	low
Oba A. et al. [36]	serious	moderate	low	low	moderate	low	low
Tanaka K. et al. [13]	serious	moderate	low	low	low	low	low
Owen JW et al. [37]	moderate	low	low	critical	critical	low	low
Tani K. et al. [38]	moderate	low	low	low	moderate	low	low
van Vledder MG et al. [20]	moderate	low	low	low	low	low	low
Auer RC et al. [18]	moderate	low	serious	low	low	low	low
Benoist S. et al. [1]	moderate	low	low	low	moderate	low	low
Boraschi P. et al. [2]	moderate	low	serious	low	low	low	low

**Table 2:** The Newcastle–Ottawa scale.

Study	Selection				Comparability	Outcomes			Total
	Representativeness of the Exposed Cohort	Selection of the Non-Exposed Cohort	Ascertainment of Exposure	Outcome of Interest Not Present at the Start of the Study		Assessment of Outcome	Length of Follow-Up	Adequacy of Follow-Up	
Ferrero A et al. [10]	*	*	*	*	*	*	*		7/8
Kim SS et al. [4]	*	*	*	*		*	*	*	7/8
Oba A. et al. [36]	*	*	*	*	*	*	*	*	8/8
Tanaka K. et al. [13]	*	*	*	*	*	*	*	*	8/8
Owen JW et al. [37]	*	*	*	*	*		*	*	7/8
Tani K. et al. [38]	*	*	*	*	*	*	*	*	8/8
van Vledder MG et al. [20]	*	*	*	*	*	*		*	7/8
Auer RC et al. [18]	*		*	*			*	*	5/8
Benoist S. et al. [1]	*	*	*	*	*	*		*	7/8
Boraschi P. et al. [2]	*		*	*		*			4/8

* One point awarded for each item.

**Table 3:** Characteristics of the studies included in the systematic review.

#	Author	Time Period	Country	# of PTS	Imaging	Primary Outcome		
						Recurrence/Viable Tumor	Survival	
1	Ferrero A et al. [10]	2004–2008	Italy	33	CT, MRI, PET	41/67 (61.2%)	NA	
2	Kim SS et al. [4]	2010–2012	Republic of Korea	36	MRI	22/158 (13.9%)	NA	
3	Oba A. et al. [36]	2010–2015	Japan	59	MRI, U/S	136/275 (49.5%)	NA	
4	Tanaka K. et al. [13]	1992–2007	Japan	23	CT	22/72 (30.6%)	≥1 CPR lesions OS_1y_: 100% OS_5y_: 69.7% DFS_1y_: 58.2% DFS_5y_: 41.7%	No CPR lesions OS_1y_: 88.5% OS_5y_: 8.9% DFS_1y_: 19.6% DFS_5y_: 0%
5	Owen JW et al. [37]	2008–2014	USA	23	CT, MRI, PET	42/72 (58.3%)	All DLMs removed DFS: 470 days	Lesion left behind DFS: 329 days
6	Tani K. et al. [38]	2010–2014	Japan	20	MRI, U/S	71/110 (64.5%)	NA	
7	van Vledder MG et al. [20]	2000–2008	USA	40	CT, MRI, PET/CT	62/112 (55.4%)	All DLMs removed OS_1y_: 93.1% OS_3y_: 58.5% OS_5y_: 37.5%	Lesion left behind OS_1y_: 93.8% OS_3y_: 63.5% OS_5y_: 63.8%
8	Auer RC et al. [18]	2000–2003	Canada	39	MRI, U/S, CT, PET	63/118 (53.4%)	OS_3y_: 85% DFS_3y_: 44%	Predicted OS_5y_: 65%
9	Benoist S. et al. [1]	1998–2004	France	38	CT	55/66 (83.3%)	OS_1y_: 100%	
10	Boraschi P. et al. [2]	2016–2020	Italy	15	CT, MRI	15/40 (37.5%)	NA	

PTS: patients, CPR: complete pathological response, DLMs: disappearing liver metastases, GA-MRI: gadoxetic acid-enhanced MRI, CT: computed tomography, MRI: magnetic resonance tomography, U/S: ultrasound, PET: positron emission tomography/computed tomography, EOB-MRI: gadolinium-enhanced MRI, CE-IOUS: contrast enhanced intraoperative U/S, NA: data not available, OS: overall survival (in years), DFS: disease-free survival.

**Table 4:** Patient demographics.

Study		Gender		Tumor Location		Pre-CTx Lesions	DLMs	Extrahepatic Lesions
#	Author	Male	Female	Colon	Rectum			
1	Ferrero A et al. [10]	25 (75.8%)	8 (24.2%)	21 (63.6%)	12 (36.4%)	153	67 (43.8%)	7 pts (21.2%)
2	Kim SS et al. [4]	28 (77.8%)	8 (22.2%)	15 (41.7%)	21 (58.3%)	289	168 (58.1%)	NA
3	Oba A. et al. [36]	42 (71.2%)	17 (28.8%)	29 (49.2%)	30 (50.8%)	NA	275	NA
4	Tanaka K. et al. [13]	17 (73.9%)	6 (26.1%)	13 (56.5%)	10 (43.5%)	472	86 (18.2%)	7 pts (30.4%)
5	Owen JW et al. [37]	15 (65.2%)	8 (34.8%)	NA	NA	200	77 (38.5%)	1 pts (4.3%)
6	Tani K. et al. [38]	10 (50%)	10 (50%)	13 (65%)	7 (35%)	619	111 (17.9%)	NA
7	van Vledder MG et al. [20]	NA	NA	NA	NA	NA	126	4 (10%)
8	Auer RC et al. [18]	29 (74.4%)	10 (25.6%)	24 (61.5%)	15 (38.5%)	166	118 (64.5%)	8 pts (27.6%)
9	Benoist S. et al. [1]	26 (68.4%)	12 (31.6%)	23 (60.5%)	15 (39.5%)	183	66 (36.1%)	NA
10	Boraschi P. et al. [2]	NA	NA	NA	NA	NA	40	NA

CTx: chemotherapy, DLMs: disappearing liver metastases, NA: data not available, PTS: patients.

**Table 5:** Statistics of detectable DLMs that were surgically treated.

#	Author	Total DLMs	DLMs Detected and Removed	Viable Carcinoma	CPR	RFA
1	Ferrero A et al. [10]	67	45	33 (73.3%)	12 (26.7%)	0 (0%)
2	Kim SS et al. [4]	168	8	0 (0%)	8 (100%)	0 (0%)
3	Oba A. et al. [36]	275	165	127 (77%)	38 (23%)	0 (0%)
4	Tanaka K. et al. [13]	86	31	11 (35.5%)	6 (19.4%)	14 (45.2%)
5	Owen JW et al. [37]	77	36 *^,^**	21 (58.3%)	10 (27.8%)	0 (0%)
6	Tani K. et al. [38]	111	68 *	52 (76.5%)	15 (22.1%)	0 (0%)
7	van Vledder MG et al. [20]	126	69	36 (52.2%)	19 (27.5%)	14 (20.3%)
8	Auer RC et al. [18]	118	68 **	44 (64.7%)	24 (35.3%)	0 (0%)
9	Benoist S. et al. [1]	66	20	20 (100%)	0 (0%)	0 (0%)
10	Boraschi P. et al. [2]	40	4 **	3 (75%)	1 (25%)	0 (0%)

DLMs: disappearing liver metastases, CPR: complete pathologic response, RFA: radiofrequency ablation. * This study contains non-specified pathology reports. ** In this study, it is not clarified whether removed tumors were detected or not and were classified as “detected”.

**Table 6:** Statistics of undetectable DLMs that were surgically treated.

#	Author	Total DLMs	DLMs Undetected and Removed	Viable Carcinoma	CPR
1	Ferrero A et al. [10]	67	12	2 (16.7%)	10 (83.3%)
2	Kim SS et al. [4]	168	0	0 (0%)	0 (0%)
3	Oba A. et al. [36]	275	68	3 (4.4%)	65 (95.6%)
4	Tanaka K. et al. [13]	86	28	0 (0%)	28 (100%)
5	Owen JW et al. [37]	77	*	*	*
6	Tani K. et al. [38]	111	10	8 (80%)	2 (20%)
7	van Vledder MG et al. [20]	126	12	5 (41.7%)	7 (58.3%)
8	Auer RC et al. [18]	118	*	*	*
9	Benoist S. et al. [1]	66	15	12 (80%)	3 (20%)
10	Boraschi P. et al. [2]	40	*	*	*

DLMs: disappearing liver metastases, CPR: complete pathologic response. * In this study, it is not clarified whether removed tumors were detected or not and were classified as “detected”.

**Table 7:** Statistics of DLMs that were left unresected and followed up.

#	Author	Total DLMs	Unresected DLMs	Recurrence	No Recurrence	Follow-Up “Mean (Range)”
1	Ferrero A et al. [10]	67	10	6 (60%)	4 (40%)	≥1 year
2	Kim SS et al. [4]	168	150 *	22 (14.7%)	128 (85.3%)	22.1 months (2.4–73.8)
3	Oba A. et al. [36]	275	42	6 (14.3%)	36 (85.7%)	27 months (9–72)
4	Tanaka K. et al. [13]	86	27	11 (40.7%)	16 (59.3%)	5 years
5	Owen JW et al. [37]	77	41	21 (51.2%)	20 (48.8%)	1 year
6	Tani K. et al. [38]	111	33	11 (33.3%)	22 (66.7%)	27.2 months (7.3–56.7)
7	van Vledder MG et al. [20]	126	45	21 (46.7%)	24 (53.3%)	20 months (7–88)
8	Auer RC et al. [18]	118	50	19 (38%)	31 (62%)	≥1 year
9	Benoist S. et al. [1]	66	31	23 (74.2%)	8 (25.8%)	≥1 year
10	Boraschi P. et al. [2]	40	36	12 (33.3%)	24 (66.7%)	8.8 months

DLMs: disappearing liver metastases. * This study includes patients with 10 DLMs who were treated with chemotherapy and remained unclassified.

## Data Availability

All the material used for the writing of the present manuscript is available upon request.
